# Current Clinical Trials to Treat Anxiety Disorders in the Elderly: A Registry-Based Review

**DOI:** 10.3390/ph19060891

**Published:** 2026-06-04

**Authors:** Gunnar P. H. Dietz, Matthias W. Riepe

**Affiliations:** 1 University of Göttingen Medical School, 37075 Göttingen, Germany; 2 Division of Geriatric Psychiatry, Ulm University, 89312 Günzburg, Germany

**Keywords:** anxiety disorder, elderly, clinical trials, drug discovery

## Abstract

**Background/Objectives**: Anxiety disorders in people over 65 y of age are common. Treatment of those disorders is often based on studies involving much younger patients. Experience shows that those treatments are regularly ineffective in the elderly, due to differences in physiology and the disparate etiology of the disease. Here, we examine current trends in research to generate data for evidence-based approaches to treat anxiety disorders in the elderly. Our objective was to evaluate the scope, methodological characteristics, and therapeutic focus of current clinical trials for anxiety disorders in the elderly, and to determine whether the existing evidence pipeline is likely to meet the substantial unmet need for effective and well-tolerated treatments. **Methods**: We searched clinicaltrials.gov for studies addressing “Anxiety disorder” and related readouts and selected those studies that included patients older than 65 y, and that had anxiety measures as primary or secondary endpoints. **Results**: We find that over 99% of clinical “anxiety” trials exclude patients older than 65 y. Sixty-six trials fulfilled our inclusion criteria. Trials specifically recruiting the elderly are a rare exception. Unexpectedly, only 10 “anxiety” trials are sponsored by the pharmaceutical industry, despite the potential rewards in such investments. **Discussion and Conclusions**: Although most clinical trials are registered in clinicaltrials.gov., our work is limited by the fact that not all clinical trials carried out world-wide are included in that database. Our findings indicate that ongoing clinical research supporting evidence-based recommendations for the treatment of anxiety in the elderly is scarce. Detailed secondary analysis of clinical trial results for the efficacy and safety of anxiolytics in various age cohorts may at least be a useful instrument for hypothesis generation, to trigger additional clinical research specifically designed to address anxiety treatment in the elderly.

## 1. Introduction

Anxiety disorders remain a pervasive source of disability across the lifespan and are associated with considerable functional impairment and adverse health outcomes in older adults, including increased risk of cognitive decline, medical comorbidity, and elevated healthcare utilization. Recent reviews indicate that clinically significant anxiety affects approximately one in ten older adults and tends to be under-recognized and undertreated compared with younger populations [1].

Treatment response differs between younger and older individuals with anxiety disorders. For example, comparative analyses of collaborative care interventions indicate that treatment benefits observed in younger adults are often attenuated or less durable in later life [2]. Likewise, evidence from cognitive–behavioral therapy trials shows modest short-term benefits in older adults but may not be more effective than alternative treatments [3]. Despite this, clinical practice frequently extrapolates therapeutic strategies established in younger cohorts to elderly patients, despite differences in comorbidity profiles, age-associated pharmacokinetics, and psychosocial contexts that influence both efficacy and safety [1].

Compounding these challenges, systematic reviews have documented a scarcity of high-quality randomized evidence specific to treatment-resistant anxiety in older adults, with few or no trials designed primarily for elderly populations [4]. For both pharmacological and psychological interventions, current data are limited in scope and generalizability, particularly for outcomes most relevant to geriatric patients, such as functional status, tolerability, and long-term remission.

In the context of Western population aging and globally increasing numbers of older adults with anxiety disorders, this gap represents a growing public health concern. Recent global burden analyses show a rising incidence and prevalence of anxiety disorders in elderly populations over recent decades, reflecting demographic shifts and persistent unmet needs [5]. Optimizing care for late-life anxiety thus requires rigorous evaluation not only of treatment effects but also of whether current research investment aligns with the scale of unmet clinical need.

The present review systematically examines ongoing clinical trials addressing the efficacy and safety of treatments for anxiety disorders in older adults and critically evaluates the extent to which research efforts are commensurate with the challenges posed by aging populations in Western societies. Our goal was to identify current research trends for anxiety disorders in older adults with respect to their scope, methodology, and therapeutic focus, and we assessed whether the existing evidence pipeline is likely to address the unmet need for effective and well-tolerated treatments. It may provide guidance with respect to where additional resources for future clinical research should be invested.

## 2. Results

### 2.1. Identification and Characteristics of Trials Addressing Anxiety in the Elderly

**Figure 1:**
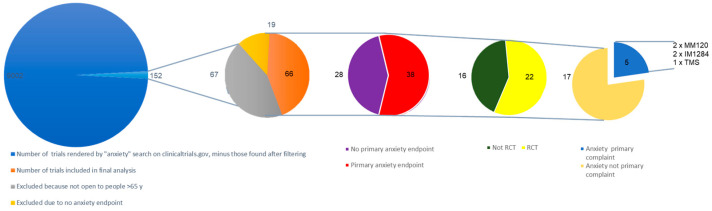
Substantially less than 1% of the “anxiety” trials registered in clinicaltrials.gov include elderly patients. In most of the 66 trials that do formally allow the recruitment of older patients, those over 65 y represent a very small fraction of the trial participants. Of those 66 trials, only 38 have primary anxiety endpoints. Of those 38, 22 follow a randomized, controlled, double-blind design. Of those 22, only 5 trials recruit patients with a primary anxiety complaint. In two of the five trials, MM120 (LSD-D-Tartrate) is tested; in two trials, ITI-1284 (5-HT_2A_ R antagonist) is used; and in one trial, TMS (transcranial accelerated intermittent theta burst stimulation) is applied. Among the 66 included trials, only 3 are carried out exclusivly in the elderly.

The initial database search of clinicaltrials.gov rendered 9002 hits, of which 152 included patients over, or equal to, 65 y of age. Of those, 19 were excluded because anxiety symptoms were not clearly among the primary or secondary readouts for the trial; 67 were excluded because patients *older* than 65 were excluded (Figure 1; Supplementary Tables S1–S3; Supplementary Figure S1).

Most of the remaining trials recruit among mixed-age populations, while only three trials specifically examine elderly patients only (Supplementary Tables S1 and S2). For most of the other trials, is it likely that elderly patients, although formally not excluded, represent only a minor fraction of the recruited study population.

Twenty-one of those trials recruit patients with GAD, or who suffer from another psychiatric disorder or personality disorder. The second largest number of trials are conducted on cancer patients (18 trials), followed by trials on surgery patients (9 trials), drug addicts (6 trials), palliative care patients (3 trials), pain patients (3 trials), neurodegenerative disease (2 trials), or others (4 trials) (Supplementary Tables S1 and S2).

**Figure 2:**
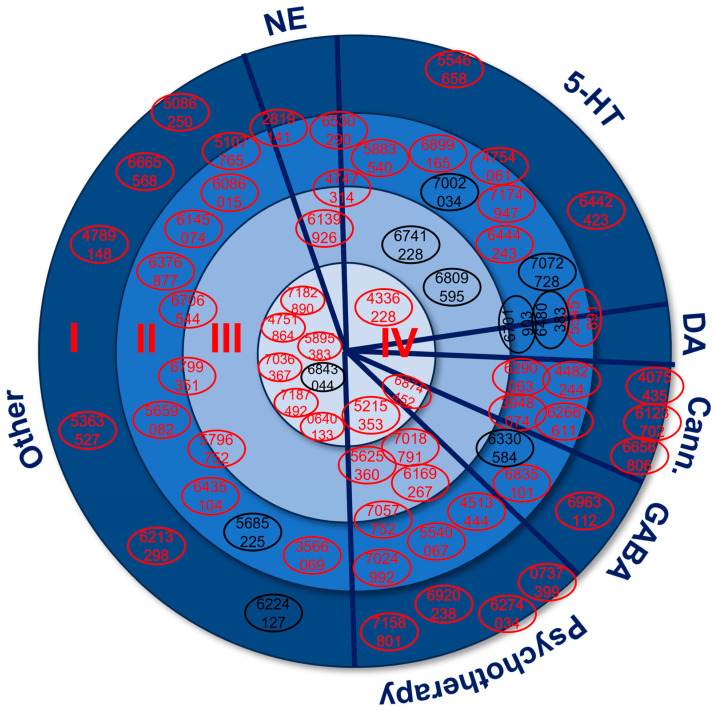
“Anxiety” clinical trials that include elderly patients identified in clinicaltrials.gov. Numbers in the outer circle indicate trials in phase 1 or in early phase 1; the inner circle represents clinical trials in phase 4; and the two circles between them showing clinical trials in phase 2 or 3, or phase 1/2, or phase 3/4. To save space, only the last seven digits of the clinical trial identifiers are indicated, i.e., “NCT0” is omitted. Trials with an academic sponsor are shown in red ink, while those with an industry sponsor are shown in black. Substances targeting signaling pathways involving norepinephrine (NE) serotonin (5-HT); dopamine (DA); gamma-aminobutyric-acid (GABA); melatonin, or other pathways (such as oxytocin, NMDA; or melatonin (“other”)) are indicated.

**Figure 3:**
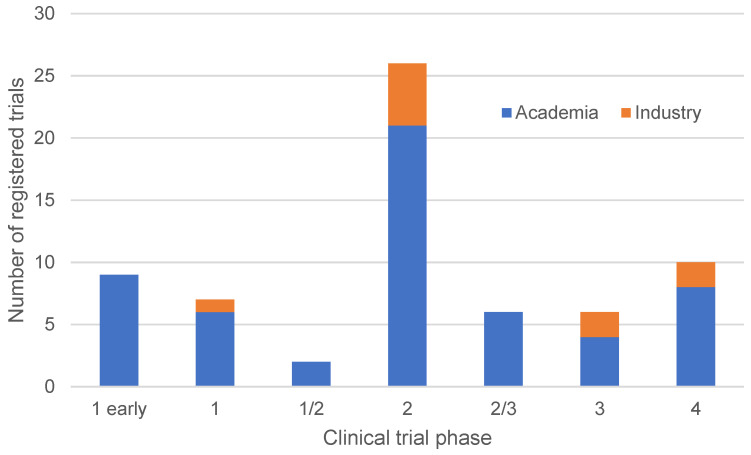
Only 10 out of 66 “Anxiety” trials in the elderly are sponsored by the pharmaceutical industry.

Most of the current trials related to anxiety are in phase 2 (34 trials in phase 1/2; 2; 2/3; Figure 2 and Figure 3). Sixteen trials are in early phase 1 or in phase 1. Six trials are in phase 3. Ten trials are in phase 4.

A frequently addressed pathway involved serotonin signaling (Supplementary Tables S1 and S2, Figure 2). Other drugs target the cannabinoid receptor, or modify signal transduction by dopamine, GABA, norepinephrine, oxytocin, NMDA, histamine, and others. In 12 trials, psychotherapeutics approaches are being tested.

Nineteen trials involved non-pharmacologic approaches, including some form of psychotherapeutic intervention of deep-brain stimulation. Most drugs tested in the 66 selected trials have been known for years, including: psilocybin (6 trials); cannabis derivatives (6 trials); LSD derivatives (3 trials); midazolam (3 trials); ketamine (2 trials); ozone treatment (2 trials); dexmedetomidine (2 trials); naltrexone/acetaminophen (2 trials); oxytocin (2 trials); fluoxetine (1 trial); propranolol (1 trial); aripiprazol (1 trial); alprazolam (1 trial); melatonin (1 trial); nitrous oxide (1 trial); memantine (1 trial); mirtazapine (1 trial); pioglitazone (1 trial); endotoxin (1 trial); and kava (1 trial). Newer compounds tested include RE104 (1 trial), Deulumateperone (ITI-1284, 2 trials), and Ranquilon (1 trial).

Unexpectedly, only 10 trials are sponsored by the pharmaceutical industry in the area examined here (Figure 3).

### 2.2. Characteristics of Trials Including Anxiety Measures as Primary Endpoints

Thirty-eight of the selected trials had anxiety measures as a primary endpoint (Supplementary Table S1); the remaining 28 among the 66 selected trials (Supplementary Table S2) had anxiety as a secondary endpoint or were a phase one trial related to anxiety, but without an anxiety endpoint at this phase.

Among the trials with an anxiety measure as a primary endpoint (Supplementary Table S1), only 22 were randomized, controlled, and blinded (Supplementary Table S1). In only 5 of those 22 trials, anxiety disorder was the primary complaint. Five additional trials were conducted in the context of cancer; in two trials, dyspepsia was the complaint. In one trial, anxiety was secondary to another end-stage fatal disease or was secondary to other diseases, including Parkinson’s disease, dyspepsia, obesity, arginine/vasopressin deficiency, and others.

## 3. Discussion

Examining the largest database for ongoing clinical trials, we find that the current clinical research pipeline is unlikely to change the existing evidence gap in the near future.

Only randomized controlled trials provide robust evidence for the efficacy and safety of a treatment, as they minimize bias, control for confounding factors, and permit causal inference regarding efficacy and safety. Here, we find that, of the 66 identified trials, only 22 are randomized and controlled and have clear measures of anxiety as their primary endpoint. Thus, these are the only ones that may eventually provide reliable evidence after trial completion, rather than being hypothesis generating.

Only six are in phase 3, suggesting that little definite additional evidence on new approaches to treat anxiety in the elderly will be available within the next years.

Moreover, clinicaltrials.gov includes highly heterogeneous clinical contexts under the broad umbrella of anxiety trials, including studies related to other psychiatric disorders or personality disorders, cancer care, pain, palliative medicine, surgery, drug use disorders, neurodegenerative disease, and other medical conditions in which anxiety may represent only a secondary symptom or exploratory endpoint. This heterogeneity substantially complicates interpretation of the findings and further weakens the knowledge gain expected to support decision-making for the geriatric psychiatrist aiming to treat anxiety in the elderly. The lack of randomized clinical trials specifically designed for the treatment of anxiety disorders in geriatric patients constrains clinical decision-making in a population largely characterized by multimorbidity, polypharmacy, and altered pharmacokinetics compared to younger patients. Some of those diseases in the elderly can trigger or exacerbate anxiety symptoms, complicate diagnostic attribution, and influence treatment response. Moreover, elderly patients are often more vulnerable to adverse drug reactions, which is exacerbated by drug–drug interactions due to the multiple medications that elderly people often take. Thus, these specific factors reduce the generalizability of clinical evidence gained from the treatment of younger patients. Considering the details of the currently registered clinical trials examined here, a substantial improvement of the situation cannot be expected any time soon. On the positive side, of the trials identified here (Supplementary Tables S1 and S2), some test new concepts that have, not that long ago, merely been assessed using preclinical models. For instance, the effect of the gut–brain axis on anxiety [6] are addressed in NCT07182890 and NCT07187492 (cf. Supplementary Tables S1 and S2). Moreover, it has been recognized that a complex disorder, like anxiety, can often not be efficiently treated by targeting just one signal transduction principle, as has been postulated evaluating preclinical data [7]. Consistent with that assumption, many treatments in clinical trials employ the interference with various neurotransmitter systems (Figure 2), e.g., acting on a combination of serotonin and dopamine (e.g., NCT05545891) the cannabinoid receptor and other receptors (e.g., NCT04075435, NCT04482244, NCT06123702, NCT06266611, NCT06656806, NCT03948074, NCT06290063), the NMDAR with other receptors, norepinephrine with dopamine and histamine and serotonin (e.g., NCT06530290), and others.

By contrast, relatively few trials evaluate truly novel compounds: RE104 (one trial by Reunion Neuroscience Inc., non-selective 5-HT-R agonist); Deulumateperone (ITI-1284, two trials, Intra-Cellular Therapies, Inc., 5-HT_2A_ R antagonist; moderate affinity for D1, D2, D4 dopamine R); and Ranquilon (one trial by Valenta Pharm JSC; Cholecystokinin receptors antagonist). The underlying biological targets of these agents have been known for many years, indicating that the majority of pharmacological approaches in current clinical development are based on earlier foundational research rather than recent mechanistic advances.

The limitations of our study include the use of only the world-wide largest registry, clinicaltrials.gov as a source of information. However, as has been pointed out by Cummings et al. [8], the US Food and Drug administration requires that all clinical trials with at least one site of patient recruitment in the US, or using a drug that is produced in the US, must be registered on clinicaltrials.gov. Moreover, most journals require the trial registration number for trial results to be revealed, providing a further incentive to register trials on that site. Thus, even though not all trials in the world are registered on clinicaltrials.gov, trends in clinical research can be derived from the trials registered here. However, we cannot exclude the possibility that, e.g., other trends may have become obvious, had we, for instance, also consulted the European Union clinical trials information system or a registry of the World Health Organization.

In our work presented here, we have partially assessed the quality of the registered trials, in that we examined whether they follow a randomized, controlled, and double-blind design. Twenty-two trials using anxiety assessment fulfil these criteria. The registry does not provide sufficiently detailed information to provide an in-depth quality assessment for each trial at this point. For instance, we have not examined whether assumptions used to calculate the power of the trial and the number of patients included are reasonable, and whether the endpoints of the trials are the most meaningful that could be selected. The trials included here have been reviewed by an ethics committee and have gone through the approval procedure. However, they have not been subject to a peer-review process, as it is common for publications in scientific journals. The depth of the conclusions that can be drawn when trial results become available is thus not clear at this point, which will, moreover, also depend on the trial outcomes. The objective of our analysis was to understand current research trends pursued to improve evidence-based approaches for anxiety treatment in the elderly, which can be addressed consulting clinicaltrials.gov. However, for most of the trials examined here, we do not know how many elderly patients will actually be recruited. For instance, in clinical trial NCT06224127, recruiting alcohol abusers aged 18–75 y of age, and trial NCT07024992, recruiting HIV-infected smokers ≥ 18 y, the majority might be below 65 y of age, while in trial NCT06530290, recruiting Parkinson’s disease patients, a clear majority will be at an advanced age. Thus, we do not know, at this point, what the patient age structure in the trials examined here will be. Although in those 66 trials retrieved here, patients above the age of 65 are admitted, it is possible that only very few people from that age group will be recruited. Of course, it is unknown which of the trials will render a positive outcome, as clinical trials fail more often than not. In conclusion, we might find that within 5 years, we are left with the same knowledge about anxiety treatment in the elderly as we have today.

The prevalence of anxiety symptoms is almost one out of five older people [9], with prevalence of anxiety in subgroups of older persons with dementia being up to about 60% [10]. Thus, it would be more than appropriate to design trials for anxiety treatment considering older persons in general or subgroups of older persons.

To improve the current gap in the evidence-based treatment of anxiety in the elderly, it is useful to speculate why there are so few studies examining the efficacy and safety of anxiety treatment in the elderly, and why the pharmaceutical industry seems to invest so few resources in that age cohort. Clinical trials consume huge amounts of resources; usually, those having a stake in such trials want a positive return on investment. With a higher variability in the patient population, the likelihood of a statistically significant effect of the treatment diminishes. Alternatively, more patients need to be included. With an average cost of USD 3.9, 13.9, and 19.2 million for clinical phase 1, 2, and 3 trials in CNS indications [11], an “unnecessary” increase in cost due to higher patient numbers is sought to be avoided.

Moreover, the older the included patients are, the more likely is the occurrence of (severe) adverse events (AE). Even if no statistically significant difference in the rate of AEs between the treatment and control groups is detected, events that occur with some frequency or degree of seriousness still need to be reported in the product labeling. This is often a requirement for drug approval by regulators, like the FDA in the United States, or by the EMA in Europe. Thus, those financing the trial tend to avoid including the most vulnerable population groups. Unfortunately, those vulnerable groups are often also those that need evidence from their specific patient cohort the most. Therefore, we suggest an in-depth discussion whether legislation could be altered to promote clinical trials in the elderly, without compromising patient safety.

To provide further incentives to conduct clinical trials in the elderly, approval of a drug should only be provided for the patient groups included in the trials that led to the approval. As older patients constitute an increasing percentage of our aging societies, and as those patients are more prone to neuropsychiatric symptoms, such a policy change might lead to more trials specifically recruiting elderly patients.

In the absence of adequately powered clinical trials specifically targeting anxiety and related neuropsychiatric symptoms in older adults, secondary meta-analytic approaches based on existing trial data may provide a pragmatic interim strategy. Such analyses, focusing on populations aged ≥65 y, could help to generate preliminary evidence regarding the efficacy and safety of current interventions and identify potentially promising or ineffective treatment approaches. However, the interpretability of these analyses is inherently limited by the marked heterogeneity in the diagnostic spectrum of anxiety across the lifespan.

In younger and middle-aged populations, anxiety symptoms are typically conceptualized within primary anxiety disorders, corresponding to ICD-10 categories F40–F41. In contrast, in older adults, anxiety symptoms more frequently arise in the context of underlying organic conditions (e.g., ICD-10 F06.4), including neurodegenerative, cardiovascular, or metabolic disorders. This fundamental difference in etiology suggests that anxiety in late life is often secondary rather than primary and may, therefore, be more responsive to optimization of the underlying medical condition than to standard anxiolytic treatments.

Notably, changes in the trajectory of underlying somatic or neurological diseases and their relationship to anxiety symptoms remain insufficiently characterized in the literature. Consequently, it is unclear to what extent improvements in the primary disease process translate into meaningful reductions in anxiety. Furthermore, pharmacological treatments established in younger populations may exhibit differential efficacy and safety profiles in older adults, given altered pathophysiology, multimorbidity, and polypharmacy. In some cases, such treatments may be less effective or may even exacerbate symptoms due to interactions with underlying conditions.

Taken together, these considerations underscore both the potential value and the inherent limitations of secondary meta-analytic approaches, and highlight the urgent need for targeted, mechanism-informed clinical trials in elderly populations.

## 4. Materials and Methods

### Database Search and Trial Inclusion Criteria

This work was inspired by the yearly assessment of the drug development pipeline in Alzheimer’s disease by Cummings et al. [8]. Our Figure 2 is reminiscent of a figure regularly presented in that work. For this review, no prepared study protocol was prepared or registered.

Between 1 May and 30 May 2025, GPHD searched https://clinicaltrials.gov, using *anxiety* as the only query, which by default of the database search tool uses the following synonymous search string: *“anxiety disorder” OR “anxiety disorders” OR “anxiety Diseases” OR “anxiety disease” OR “anxiety diagnosis” OR “anxiety condition” OR “anxiety other Disease” OR Anxiety OR “Anxiety NOS” OR anxious OR Anxiety Scale OR “anxiety symptom” OR “symptoms anxiety” OR anxieties OR Anxiousness OR Angst OR “Feeling anxious” OR “Anxiety reaction” OR “Reaction anxiety”*. That search query rendered 9002 results. Subsequently, the following 11 automatic search filters, provided by the database site, were applied: **Study status**: Not yet recruiting; OR Recruiting; OR Active, not recruiting; OR Enrolling by invitation AND **Eligibility Criteria**: Age Older adult (65+) AND **Study Phase**: Early Phase 1 OR Phase 1 OR Phase 2 OR Phase 3 OR Phase 4. Thus, studies were also retrieved in which, besides patients over 65, younger patients were also permitted. In total, 152 studies were retrieved using these search criteria (Supplementary Figure S1). Upon further inspection, GPHD excluded 65 studies because they did not include patients older than 65 y of age. Studies were only included if they permitted patients up to at least 70 y of age. Two studies were excluded because they included only 4–6 wks postpartum or pregnant women. Moreover, clinical phase 2, 3, and 4 trials were excluded when the primary or secondary endpoints did not include assessment of anxiety symptoms or endpoints were not sufficiently clear (18 studies) (Supplementary Table S3).

Parameters of included trials shown in Supplementary Table S1 and in Supplementary Table S2 include the full study title with a link to the corresponding web site; the trial ID; Sponsor; trial phase; number of participants and treatment groups; important aspects of the trial design; molecular target (if known), and major endpoints. Figure 2 visualizes test compounds targeting signaling pathways involving norepinephrine (NE); serotonin (5-HT); dopamine (DA); gamma-aminobutyric-acid (GABA); melatonin; cannabinoids; and others. Figure 3 indicates the phases that the trials are in, and whether the sponsor is an academic or a private institution.

The authors used a ChatGPT system based on a GPT-5–series large language model (OpenAI), accessed in February 2026 for language editing in selected parts of the Introduction. No references were generated by the AI tool. All content was critically reviewed and verified by the authors.

## 5. Conclusions

One of the unexpected outcomes of this study is the finding that researchers overall, and the pharmaceutical industry in particular, invest little resources in a field with a very high unmet need. In our aging society, substantial revenue could be generated; investments appear to be well commensurate with the potential reward. We will watch closely whether the near future will render more clinical research specifically focusing on the alleviation of anxiety in the elderly population. Until then, at least an in-depth analysis of elderly patient data available either from secondary analysis of controlled clinical trials, real-world evidence analysis, and observational studies, may provide initial hypotheses on the most auspicious treatments for anxiety in senior citizens.

## Data Availability

No new data were created or analyzed in this study.

## Supplementary Materials

The following supporting information can be downloaded at: https://www.mdpi.com/article/10.3390/ph19060891/s1, Table S1: Clinical trials retrieved from clinicaltrials.gov addressing anxiety in the elderly and with anxiety primary endpoints. Table S2: Clinical trials retrieved from clinicaltrials.gov addressing anxiety in the elderly and with anxiety secondary endpoints. Table S3: Trials detected by search query but excluded from this study. Supplementary Figure S1: Identification of studies from clinicaltrials.gov. References [12,13,14,15,16,17,18,19,20,21,22,23,24,25,26,27] can be found in supplementary materials files.

## Abbreviations

The following abbreviations are used in this manuscript:

ASEC	Antidepressant side effect checklist
BAI	Beck Anxiety Inventory self-rated score
Bid	twice per day
CGII scale	Clinical Global Impression—Improvement scale
CGI-S	Clinical Global Impression—Severity scale
GIC	global impression of change
Co	cross-over
Db	double-blind
dd	double-dummy
GAD	Generalized anxiety disorder
HADS	Hospital anxiety and depression anxiety subscore.
HAMA	Hamilton Anxiety Rating Scale
HAMD	Hamilton Depression Rating Scale
Mc	multicenter
Mono	monocentric
OCD	Obsessive-compulsive disorder
open	open label
Pc	Placebo controlled
PCL	PTSD checklist
Pg	parallel groups
Pk	pharmacokinetic
PSWQ	Penn State Worry Questionnaire
PTSD	Post-traumatic stress disorder
QOL	quality of life
R	receptor
Rd	randomized
SAD	social anxiety disorder
SADC	separation anxiety disorder of childhood
Sc	sham control
SNRI	Serotonin/noradrenaline reuptake inhibitor
SSRI	Serotonin uptake inhibitor
STAI	State–Trait Anxiety Inventory
VAS	Visual analogue scale
Wk	Week
Y	Year or years
