# Supplementary material for: Current Clinical Trials to Treat Anxiety Disorders in the Elderly: A Registry-Based Review
Source: Pharmaceuticals (Basel). 2026 Jun 4;19(6):891. doi: 10.3390/ph19060891 (PMC13305826; doi:10.3390/ph19060891)
Supplement: Supplementary file 1 [file pharmaceuticals-19-00891-s001.zip › PRISMA_SupplementFigure Dietz.pdf]

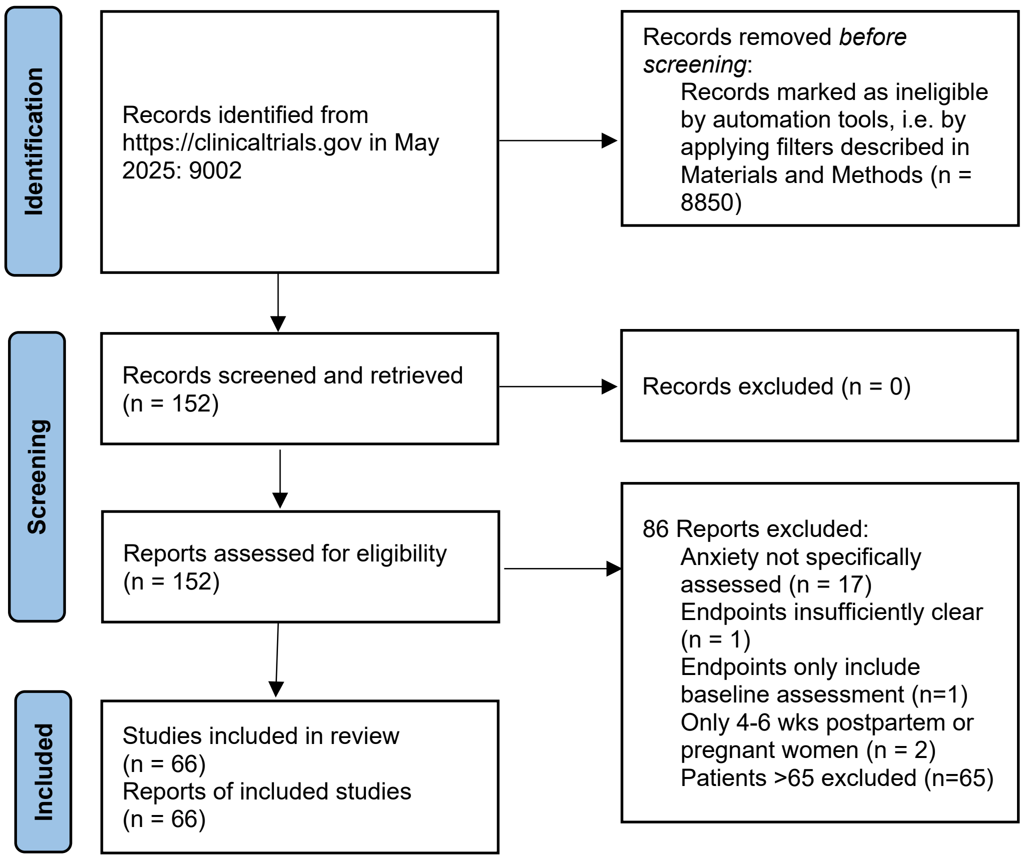

Supplementary Figure S1: Identification of studies from Clinicaltrials.gov. Source of scheme: Page MJ, et al. BMJ 2021;372:n71. doi: 10.1136/bmj.n71 [27]. The scheme is licensed under CC BY 4.0. To view a copy of this license, visit https://creativecommons.org/licenses/by/4.0/
