# Supplementary material for: Current Clinical Trials to Treat Anxiety Disorders in the Elderly: A Registry-Based Review
Source: Pharmaceuticals (Basel). 2026 Jun 4;19(6):891. doi: 10.3390/ph19060891 (PMC13305826; doi:10.3390/ph19060891)
Supplement: Supplementary file 1 [file pharmaceuticals-19-00891-s001.zip › Table S1 Analysed trials Primary Endpoint.pdf]

**Supplementary Table S1. Clinical Trials retrieved from clinicaltrials.gov addressing anxiety measures as primary endpoints, and in addition also included patients >65 y.** 2 trials with exclusive recruitment of elderly patients are marked green. Anxiety endpoints are printed in **bold**. Trial titles corresponding to a randomized, controlled, double-blind design are shown in **bold**. The order of the trials is determined by the major molecular target addressed by the treatment.

| Study title, Link | Trial ID; Sponsor | Phase | Participants | Trial Design | Treatment groups | Target | Major Endpoints |
|---|---|---|---|---|---|---|---|
| **A Phase 3 Trial of MM120 for Generalized Anxiety Disorder (Voyage)** | **NCT06741228 Mind Medicine, Inc.** | 3 | 200 GAD patients 18-74 y | Rd, db, pc, pg, mc | 12 wks 100 µg MM120 (LSD D-Tartrate) or placebo plus 40 wks open label extension | 5-HT R non-selective agonist (LSD derivative) [12] | **Primary**: **Change from Baseline in HAM-A total score at Week 12** **Secondary**: 23 different tests assessing various psychiatric and other scores |
| **A Phase 3 Trial of MM120 for Generalized Anxiety Disorder (Panorama)** | **NCT06809595 Mind Medicine, Inc.** | 3 | 250 (up to 375) GAD patients 18-74 y | Rd, db, pc, pg, mc | 12 wks 2-50 µg MM120 (LSD D-Tartrate), or 100 µg MM120, or placebo plus 40 wks open label extension | 5-HT R non-selective agonist (LSD derivative) [12] | Primary: **Change from Baseline in HAM-A total score at Week 12** Secondary: an array of 24 different tests assessing various psychiatric and other scores |
| PSilocybin for psYCHological and Existential Distress in PALliative Care (PSYCHED-PAL) | NCT04754061 Ottawa Hospital Research Institute | 1/2 | 20 patients with 1 to <12 month life expectancy with depression, anxiety, or low well being,, ≥18 y | Open, one arm,recruited at 2 hospitals in Ottawa | 1 wk 1mg psilocybin on Monday and Thursday, followed by 1 wk 1 mg/d psilocybin Monday-Friday; followed by 2 x 1 mg/d Monday-Friday; followed by 3 x 1 mg/d psilocybin Monday-Friday, for a total of 4 wks. | 5-HT R non-selective agonist [13] | **Primary**: Recruitment Rate; Intervention Completion Rate; Follow-up Completion Rate; Number of Participants With Adverse Events; Psychological Distress - **Anxiety** and Depression and Well-being; dosing evaluation. **Secondary**: Demoralization scale II; HAMD; World Health Organization Quality of Life, Brief Version; Wish to die; distress thermometer |
| Effects of Psilocybin in Obsessive | NCT05546658 Johns Hopkins University | 1 early | 30 patients 21-70 y with obsessive compulsive disorder | Rd, co, open label, mono | 1 x 20 mg x psilocybin followed by | 5-HT R non-selective agonist [13] | Primary: Yale Brown Obsessive Compulsive Scale (Y-BOCS); **State-** |

| | | | | | | | |
|---|---|---|---|---|---|---|---|
| Compulsive Disorder | | | | | another 30 mg 2 wks later | | **Trait Anxiety Inventory (STAI)**; Beck Depression Inventory II (BDI-II); Quality of Life Enjoyment and Satisfaction Questionnaire (Q-LES-Q) |
| **Psilocybin-Assisted Psychotherapy in Cancer Patients With Adjustment Disorder** | **NCT07072728 Psyence Australia Pty Ltd** | 2b | 87 cancer patients with adjustment disorder 18-80 y | Rd, db, low dose ctrl., mc | Either 25 mg or 10 mg or 1 mg PEX010 single doses day14 in addition to psychotherapy | 5-HT R non-selective agonist [13] | Primary: **HAMA**; Treatment related adverse events; Sheehan Suicide Tracking Scale |
| **Lysergic Acid Diethylamide (LSD) in Palliative Care** | **NCT05883540 University Hospital, Basel, Switzerland** | 2 | 60 patients with end-stage fatal disease ≥ 22 y | Rd, pg, db, mc | 100 µg ($1^{st}$ session) and 100 µg or 200 µg ($2^{nd}$ session) LSD or 25 µg LSD in $1^{st}$ and $2^{nd}$ session | 5-HT R non-selective agonist [12] | Primary: **Change STAI-S 2wks after $2^{nd}$ intervention** Secondary, at various additional time points: **STAI-S**; pain numeric rating scale; change in opioid use; spiritual well-being score; demoralization scale II score; quality of life assessment; Emotional Condition Rating Scale; **HAMD**, adverse events; and many other readouts |
| **Study of ITI-1284 as an Adjunctive Treatment in Patients With Generalized Anxiety Disorder** | **NCT06480383 Intra-Cellular Therapies, Inc.** | 2 | 705 GAD patients with little response to other treatment ≥18y | Rd, db, pc, mc | 6 wks either ITI-1284 10 mg/d, ITI-1284 20 mg/d, or placebo, plus 1 wk later follow-up visit | $5\text{-}HT_{2A}$ R antagonist; moderate affinity for D1, D2, D4 dopamine R | Primary: **HAM-A wk 6** Secondary: CGI-S |
| **Study of ITI-1284 as Monotherapy Treatment in Patients With** | **NCT06701903 Intra-Cellular Therapies, Inc.** | 2 | 570 GAD patients with little response to other treatment ≥18y | Rd, pc, db, mc, pg | 6 wks either ITI-1284 10 mg, ITI-1284 20 mg, or placebo, plus 1 wk later follow-up visit | $5\text{-}HT_{2A}$ R antagonist; moderate affinity for D1, D2, D4 dopamine R | Primary: **HAM-A** Secondary: CGI-S |

| | | | | | | | |
|---|---|---|---|---|---|---|---|
| **Generalized Anxiety Disorder** | | | | | | | |
| Apply tACS to Alleviate Anxiety Symptoms | NCT06086015 NeuroCognitive and Behavioral Institute Clinical Research Foundation | 2 | 40 patients >5 y with GAD, SAD, SADC, or PTSD | Rd, sc, db, cross-over, optional open label extension | 12x25 minutes 5 hz **t**ranscranial **al**ternating **c**urrent **s**timulation (tACS) within 3-4 wks | Antero-lateral amygdala | Primary: **BAI, HAMA** Secondary: PCL |
| **Examining Safety, Efficacy and Feasibility of Preoperative Propranolol in Patients With PDAC** | **NCT06145074 Zealand University Hospital** | 2 | 30 patients with suspected surgically resectable pancreatic cancer ≥ 18 y | Rd, pc, pg, db, 2 locations | 2 x 40 mg propanolol/d or placebo for 10 d leading up to surgery | Beta R; catecholamine signaling | Primary: preoperative **HAMA**, HADS, quality of life assessment; 30 d, 90 d, 1 y, 3 y, and 5 y postoperative survival; 1 y after surgery: assessment of tumor histopathology and blood sample analysis Secondary: Blood pressure and heart rate monitoring during propanonol application |
| Cannabidiol Solution for the Treatment of Behavioral Symptoms in Older Adults With Mild Cognitive Impairment or Alzheimer's Dementia | NCT04075435 Mclean Hospital | 1 early | Patients with MCI or moderate to mil Alzheimer's dementia, and anxiety 55-90 y | Single group, open label, mono | high CBD/low THC sublingual solution twice/d | Cannabinoid R modulation? Interaction with other neurotransmitters? | Primary: measure of **anxiety on neuropsychiatric inventory-clinician scal**e Secondary: **Total score Generalized Anxiety Disorder 7**; number serious adverse events; Week 8 MMSE compared to baseline MMSE; confusion assessment method; medication side effects questionnaire Reduction in agitation and aggression symptoms; Cohen-Mansfield inventory; Zarit caregiver burden interview; anxiety domain of NPI-C and GAD-7; stability of caregiver burden reduction |

| | | | | | | | |
|---|---|---|---|---|---|---|---|
| **RCT of CBD for Anxiety in Advanced Breast Cancer** | **NCT04482244 Dana-Farber Cancer Institute** | 2 | 50 Female patients with stage IV or metastatic breast cancer ≥18y | Rd, pg, db, mono | Single dose cannabidiol of unrevealed dose or placebo prior CT scan or PET | Cannabinoid R modulation? Interaction with other neurotransmitters? | Primary: Visual Analog Mood Scale, **anxiety subscale** Secondary: Common Terminology Criteria for Adverse Events; Visual Analog Mood Scale (VAMS) for eight specific mood states: Afraid, Confused, Sad, Angry, Energetic, Tired, Happy, Tense; EORTC-QLQc30 questionnaire-nausea subscale; pain intensity scale |
| **Cannabis for Palliative Care in Cancer (ARCTiC)** | **NCT06266611 University of Colorado, Boulder** | 2 | 185 Cancer patients with sleep problems, pain, depression, or anxiety ≥25 y | Rd, pg, db, mono | 8 wks either 200mg CBD+4mg THC, or 200 mg CBD, or placebo | Cannabinoid R modulation? Interaction with other neurotransmitters? | **Primary**: Patient-Reported Outcomes Measurement Information System) Pain Interference; Patient-Reported Outcomes Measurement Information System for Pain Intensity, Brief Pain Inventory survey, Sleep Disturbance 4a, Sleep-Related Impairment 4a, fatigue 4a, Pain Intensity (Right Now); Depression **Anxiety Stress Scale - 21 Item (DASS-21)**; Health Related Quality of Life Short Form 12 (SF-12); Functional Assessment of Cognitive Function - Cognitive; Stroop Task; Digit Symbol Substitution Task; Conners Continuous Performance Test - Version 3; Plasma cannabinoid levels; Drug Effects Questionnaire (DEQ); Addiction Research Center Inventory - Marijuana Scale; Profile of |

| | | | | | | | |
|---|---|---|---|---|---|---|---|
| | | | | | | | Mood States; acute plasma cannabinoid levels. Secondary: Use of medication for pain, psychiatric conditions, sleep |
| **Cannabidiol and Older Adult Cannabis Users** | **NCT06290063 University of Colorado, Boulder** | 2 | 385 Cannabis users currently taking medication for pain, sleep, or mood >59y | Rd, pc, pg, db, mono | 200mg CBD/4mg THC capsules, or 200 mg CBD only, or placebo | Cannabinoid R, TRP R, 5-$HT_{1A}$ R, L-type $Ca^{2+}$; PPARγ, [14] | Baseline, 4, 8 wks: Primary: 16 different mostly motor, cognitive or psychiatric tests including **anxiety** **Secondary**: Polypharmacy for pain, sleep, psychiatric symptoms; sleep quality, chair stand task |
| Efficacy and Safety of Ranquilon in Patients With Anxiety Disorders Due to Neurasthenia and Adaptation Disorders | **NCT06843044 Valenta Pharm JSC** | 4 | 250 patients 18-70 y with anxiety disorders due to neurasthenia and adaptation disorders | Rd, open label, reference-controlled, mc | 28 d 6 mg Ranquilon/d or 30mg/d Afobazole | Cholecystokinin receptors antagonist | Primary: **50% or more reduction in HAMA compared to baseline** Secondary: 53 various measurements assessing anxiety, GCI-S, fatigue, suicide risk, stress measures, safety and tolerability |
| **Treating Stress and Anxiety in Individuals With Alcohol Use Disorder (AUD)** | NCT06224127 Soovu Labs Inc. | 1 | 36 patients with AUD 18-75 y old | Rd, db, active or mock treatment, mono | Heating of skin with heating pods, or tactile stimulation, single treatment with respective mock treatment, or combination of both active treatments | CT fiber stimulation | Primary: **anxiety short form; visual analogue scale for anxiety**; desire for alcohol questionnaire Secondary: Oxytocin and cortisol levels |
| **Administration of Intranasal Midazolam for Anxiety in Palliative Care** | **NCT06330584 Insel Gruppe AG, University Hospital Bern** | 2 | 36 palliative patients ≥18y with acute anxiety | Rd, pc, db, pg, mc | 0 mg; 0.45mg; or 0,9 mg intranasal Midazolam | $GABA_A$ R positive allosteric modulator [16] | Primary: **0 min and 30 min after intervention VAS on anxiety** Secondary: Richmond Agitation Sedation Scale Palliative Version (RASS-PAL); required doses during 24 h; vital signs; pk data |

| | | | | | | | |
|---|---|---|---|---|---|---|---|
| Perioperative Music in Obese Patients Under Spinal Anesthesia | NCT06835101 Asklepieion Voulas General Hospital | 2 | 40 obese patients undergoing surgery requiring spinal anesthesia ≥18 y | Rd, pg, db, mc | Either music before and to the end of surgery, or sedative midazolam | $GABA_A$ R positive allosteric modulator [16] | Primary: **Spielberger State-Trait Anxiety Inventory** after spinal anesthesia Secondary: **Spielberger State-Trait Anxiety Inventory** after surgery; Time needed to perform spinal anesthesia; patient satisfaction 24 h after surgery; patient-anesthesiologist communication efficacy; adverse events |
| **Intraoperative Midazolam on Delirium Outcome of Elderly Patients** | **NCT06963112 Chinese PLA General Hospital** | 1 | 612 surgical patients 65-85 y old | Rd, pc, db, pg, mc | 2 mg midazolam or placebo injection for anesthesia induction | $GABA_A$ R positive allosteric modulator [16] | Mostly postoperative days 1 – 7: **Primary**: Confusion Assessment Method; **Generalized Anxiety disorder-7 scale** **Secondary**: **GAD-7 scale for anxiety severity;** Resting and movement pain scores using NRS scale; Onset time of delirium episodes; Frequency of delirium episodes; Subtype of delirium episodes; Intraoperative awareness of the occurrence; Incidence of postoperative nausea and vomiting; Incidence of delayed extubation; Incidence of non delirium complications and all-cause mortality; Length of hospital stay; 1-Year mortality |
| **Efficacy of Clostridium Butyricum in** | **NCT07182890 Xijing Hospital of Digestive Diseases** | 4 | 180 patients with functional dyspepsia and anxiety and depression 18-80 y | Rd, pg, db, pc, | 3 x 1260 mg/d Clostridium butyricum or placebo for 4 wks | Gut-brain axis | Primary: **Hospital Anxiety** and Depression **Scale** Secondary: overall treatment effectiveness |

| | | | | | | | |
|---|---|---|---|---|---|---|---|
| Alleviating Anxiety and Depression in Patients With Functional Dyspepsia | | | | | | | evaluation questionnaire; global overall symptom score; Short-form Nepean Dyspepsia Index; Pittsburgh Sleep index |
| Efficacy of Bacillus Coagulans in Alleviating Anxiety and Depression in Patients With Functional Dyspepsia | **NCT07187492 Xijing Hospital of Digestive Diseases** | 4 | 180 patients with functional dyspepsia and anxiety and depression 18-80 y | Rd, pg, pc, db | 3 x 1050 mg/d Bacillus coagulans or placebo for 4 wks | Gut-brain axis | Primary: **Hospital Anxiety and Depression Scale** Secondary: overall treatment effectiveness evaluation questionnaire; global overall symptom score; Short-form Nepean Dyspepsia Index; Pittsburgh Sleep index |
| The Use of Sublingual Melatonin Premedication in Geriatric Cataract Surgery | **NCT07036367 Al-Azhar University** | 4 | 60 patients before unilateral cataract surgery under local anesthesia ≥ 60 y | Rd, pc, db, pg, mono | 3 mg sublingual melatonin or placebo 60 min before surgery | Melatonin R | Primary: Perioperative **VAS for anxiety 30 and 60 min before surgery and 15 and 30 min after anesthesia** |
| Brain Stimulation & Generalized Anxiety Study | **NCT04751864 Proof Pilot** | 4 | 150 GAD patients ≥21 y | Rd, crossover, mono | 8 wks, 2 bid 20 min Fisher Wallace Cranial Electrotherapy Stimulator (CES) active, or 4 wks sham, then crossover assignment to active for the former sham group for another 4 wks | Modulation of brain waves? | Primary: **Change in BAI at week 4** Secondary: Change in Promise Sleep-SD; change in patient health questionnaire; **change BAI wk 4 vs wk 8**; SAFTEE self-reported side effect measured. |
| Connectomic Targeted TMS Target for Refractory Anxiety | **NCT06376877 Brigham and Women's Hospital** | 2 | 80 patients 18 – 70 y with various anxiety-related disorders | Rd, db, sham-controlled, mono | 50 treatments over 5 d Transcranial accelerated intermittent theta burst stimulation Magenetic stimulation | Modulation of brain waves? | Primary: **BAI self-report scale 1 wk and 1 month after treatment Secondary: 11 tests assessing various scores** |

| | | | | | | | |
|---|---|---|---|---|---|---|---|
| | | | | | (aiTMS) or sham (the latter offered open-label crossover extension) | | **for anxiety** and other psychiatric symptoms |
| **Evaluating the Effect of Mirtazapine on Anxiety in Parkinson's Disease Patients** | **NCT06530290 Leila Dargahi, Shahid Beheshti University of Medical Sciences** | 2 | 64 patients ≥18 y with anxiety and mild to moderate Parkinson's disease | Rd, pc, db, pg, mono | 12 wks 12 mg mirtazapine/d or placebo | Norepinephrine and dopamine effect by α2-adrenergic R inhibition; Histamine-1 (H1) R antagonist, 5-HT2A, 5-HT2C, 5-HT3 R. moderate to weak antagonist peripheral α1-adrenergic and muscarinic receptors [19] | All at 4 and 12 wks treatment: Primary: **HAM-A, Parkinson Anxiety Questionnaire (PAS)** Secondary: HAM-D; Parkinson's Disease Fatigue Scale (PDFS); Parkinson's Disease Sleep Scale (PDSS); Parkinson's Disease Quality of Life (PDQL) questionnaire |
| Self-management of Sedative Therapy by Ventilated Patients | **NCT02819141 Mayo Clinic** | 2/3 | Mechanically ventilated patients currently receiving continuous infusion of sedative or opiod ≥18 y | Rd, open label, pg, at two clinics in Minnesota | dexmedetomidine infusion or no intervention control | Norepinephrine release inhibition: α-2 adrenoceptor specific and selective presynaptic agonist | **Primary**: **VAS for changes in anxiety**; changes in duration of days receiving mechanical ventilation after study enrollment; Changes in delirium using confusion assessment method-ICU **Secondary**: Richmond agitation-sedation scale; comparison of sedative exposure. |

| | | | | | | | |
|---|---|---|---|---|---|---|---|
| Clinical Study of Dexmedetomidine Administered Intranasally to Relieve Perioperative Anxiety and Depression in Patients With Colorectal Tumors | NCT06139926 First Affiliated Hospital of Chongqing Medical University | 3 | 126 colorectal cancer patients before first oncologic procedure ≥18 y | Rd, pg, mono | Either Intranasal or iv Dexmedetomidine | Norepinephrine release inhibition: α-2 adrenoceptor specific and selective presynaptic agonist | Primary: Changes in perioperative HADS |
| Aromatherapy in the Treatment of Early Breast Cancer | NCT06435104 Sun Yat-Sen Memorial Hospital of Sun Yat-Sen University | 2 | 30 women with early breast cancer and mild anxiety 18-80y | Rd, pg, open label, mono | neoadjuvant chemotherapy with or without essential oil aromatherapy | Odorant R stimulation | Primary: **HAMA and State-trait anxiety inventory scale** Secondary: Survival after 2 y; Quality of life questionnaire C30; fraction of patients with complete pathologic response after 2 y; various other measures of chemotherapy response rate; **anxiety self-rating scale (SAS);** Pittsburg sleep quality index; HAMD |
| **Intranasal Oxytocin as Enhancer of Psychotherapy Outcomes in Severe Mental Illness** | **NCT03566069 Shalvata Mental Health Center** | 2 | Patients with various psychiatric conditions, e.g. depression, anxiety disorders, OCD, personality disorders, PTSD ≥18 y | Rd, pg, pc, db, mono | Intranasal oxytocin or placebo | Oxytocin R | Primary: **Anxiety and depression Hopkins symptoms checklist - short form** Secondary: 6 item Working Alliance Inventory self-report measurement of Adult Attachment |
| **Effects of Intranasal Oxytocin in Patients With Arginine-** | **NCT04789148 Elizabeth Austen Lawson** | 1 | 40 patients with arginine-vasopressin deficiency ≥18 y | Rd, pc, db, crossover, mono | 6 IU or 24 IU Single dose intranasal oxytocin or placebo crossover, followed by 2 wks 6 IU oxytocin 3 x/d or placebo | Oxytocin R | Primary: **Anxiety behavior dot-probe task difference between low dose and pc** Secondary: dot-probe task difference between all three interventions; Depressive behavior |

| | | | | | | | |
|---|---|---|---|---|---|---|---|
| **vasopressin Deficiency** | | | | | | | probabilistic reward task; emotion recognition task |
| Interest of Medical Hypnosis in Anxious Patients Treated by Radiotherapy | NCT04513444 Institut Claudius Regaud | 2 | 45 patients with solid malignant tumors with moderate to high anxiety during radiotherapy treatment ≥18 y | Rd, open, pg, mono | Patients with or without hypnosis during radiotherapy for 2 months | Psychotherapy | Primary: **At least 10 point decrease in Spielberger State-Trait Anxiety Inventory - State Anxiety form** Secondary: **0 to 10 anxiety scale**; European Organization for Research and Treatment of Cancer Quality of life questionnaire |
| **A Study Comparing Music Therapy and Cognitive Behavioral Therapy for Anxiety in Cancer Survivors** | NCT05215353 Memorial Sloan Kettering Cancer Center | 4 | 350 cancer patients with anxiety ≥18 y | Rd, pg, mc, investigator and outcome assessor blinded | Music therapy or cognitive behavioral therapy given virtually | Psychotherapy | Primary: **HADS** anxiety subscale after 6 wks and 26 wks |
| Improving Mental Health Among the LGBTQ+ Community | NCT05540067 Brown University | 2 | All LGBTQ+ identified patients with anxiety or depression ≥18y | Rd, pg, mono | Acceptance-Based Behavior Therapy or treatment as usual over 39 wks | Psychotherapy | Primary: **HAMA**; Quick Inventory of Depressive Symptomatology - Clinician Rating **Secondary: Generalized Anxiety Disorder-7 (GAD-7)**; Patient Health Questionnaire-9 (PHQ-9) |
| **Passive Music Intervention for the Reduction of Anxiety in Patients Undergoing Bone Marrow Aspiration and/or Biopsy** | NCT06169267 City of Hope Medical Center | 3 | 110 patients undergoing bone marrow biopsy or aspiration ≥18 y | Rd, pg, mono | Bone marrow aspiration of biopsy with or without music | Psychotherapy | Up to 4 months: Primary: **State-Trait Anxiety Inventory** Secondary: VAS pain level |
| MUSE-S Headband System for Improving Anxiety | NCT06274034 Mayo Clinic | 1 early | 40 women with breast cancer and anxiety and insomnia 18-80 y | Single group open, mono | Use of EEG headband for meditation and phone app during the day | Psychotherapy | **Primary**: Frequency of meditation use; wearability of EEG band assessed by post-study satisfaction questionnaire; |

| | | | | | | | |
|---|---|---|---|---|---|---|---|
| and Insomnia Among Breast Cancer Survivors | | | | | | | questionnaires on sleep, quality of life, **anxiety** |
| Enhancing Smoking Cessation for African American People Everywhere | NCT07024992 University of Houston | 2 | 300 black HIV-infected smokers with moderate to high anxiety sensitivity or anxiety or depression ≥18 y | Rd, pg, open label, mono | nicotine replacement with 2 different behavioral apps for smoking cessation or no app | Psychotherapy | Primary: smoking abstinence over past 7 d; **Overall Anxiety Severity Index Scale**; Overall Depression Severity Index Scale; QOL World Health Organization HIV of Life Brief Scale, the QOL Enjoyment and Satisfaction Questionnaire, the Index of Engagement in HIV Care; HIV care adherence Secondary: Short Scale Anxiety Sensitivity Index |
| Virtual Reality for Pain and Anxiety Relief During Peripheral Angioplasty | NCT07057752 Kastamonu University | 2/3 | 52 patients with peripheral arterial disease undergoing angioplasty, 18-100y | Rd, pg, open, mono | Virtual reality glasses or routing treatment | Psychotherapy | Primary, after 1 d: Pain numerical rating scale from 0 to 10; **State Anxiety Inventory-6** |
| Effect of Kava on Anxiety and Stress in Cancer Survivors | NCT06213298 Masonic Cancer Center, University of Minnesota | 1 early | 43 cancer survivor patients ≥18y | Rd, pc, open label, cross over, mono | 14 d 75 mg kava or placebo, then washout and cross over | voltage-gated $Na^+$ channels blockade [21]; blockade of $Ca^{2+}$ channels[22]; affinity for $CB_1$ R [23]; enhanced ligand binding to $GABA_AR$ | **Primary**: **Promis-29 anxiety measure**; adverse events CTCAE v.5.0 **Secondary**: none |

| | | | | | | | |
|---|---|---|---|---|---|---|---|
| | | | | | | [18]; reversible inhibition of MAO-B; norepinephrine and dopamine reuptake inhibition [25]; suppression of eicosanoid thromboxane A2 synthesis, enhancing $GABA_A$R function [26] | |

Abbreviations: ASEC: Antidepressant side effect checklist; BAI Beck Anxiety Inventory self-rated score; Bid: twice per day; CGII scale: Clinical Global Impression – Improvement scale; CGI-S Clinical Global Impression - Severity scale; GIC global impression of change; Co: cross-over; Db: double-blind; dd: double-dummie; GAD: Generalized Anxiety disorder; HADS: Hospital anxiety and depression anxiety sub-score.; HAMA: Hamilton Anxiety Rating Scale; HAMD: Hamilton Depression Rating scale; Mc multicenter; Mono: monocentric; OCD: Obsessive-compulsive disorder; open: open label; Pc: Placebo controlled; PCL: PTSD checklist; Pg: parallel groups; Pk: pharmacokinetic; PSWQ Penn State Worry Questionnaire; PTSD: Post-traumatic stress disorder; QOL: quality of life; R: receptor; Rd: randomized; SAD: social anxiety disorder; SADC: separation anxiety disorder of childhood; Sc: sham control; SNRI: Serotonin/noradrenaline reuptake inhibitor; SSRI: Serotonin uptake inhibitor; STAI: State-Trait Anxiety Inventory; VAS: Visual analogue scale; Wk: Week
