# Supplementary material for: Current Clinical Trials to Treat Anxiety Disorders in the Elderly: A Registry-Based Review
Source: Pharmaceuticals (Basel). 2026 Jun 4;19(6):891. doi: 10.3390/ph19060891 (PMC13305826; doi:10.3390/ph19060891)
Supplement: Supplementary file 1 [file pharmaceuticals-19-00891-s001.zip › Table S2 Sec Endpoint Anxiety Analysed trials.pdf]

Supplementary Table S2. Included clinical Trials retrieved from clinicaltrials.gov addressing anxiety disorder that also included patients >65 y, but that did not use anxiety assessment as a primary endpoint. Trials with exclusive recruitment of elderly patients are marked green. Anxiety endpoints are printed in **bold**. The order of the trials is determined by the major molecular target addressed by the treatment, i.e. column 7.

Study title, Link	Trial ID; Sponsor	Phase	Participants	Trial Design	Treatment groups	Target	Major Endpoints
SSRI Antidepressant Fluoxetine Improving Immunotherapy Efficacy in Advanced Hepatobiliary Malignancy Patients With Depression and Anxiety	NCT07174947 First Affiliated Hospital of Wenzhou Medical University	2	240 patients with liver or gall bladder tumors with depression and anxiety 18-80 y	Rd, pg, open	20 mg/d oral Fluoxetine or placebo	5-HT reuptake inhibition	Primary: Response Rate to concomitant cancer treatment by CT/MRI. **Secondary**: Overall survival; progression-free survival; **anxiety and depression measured by GAD-7 and PHQ-9;** European Organization for Research and Treatment of Cancer Quality of Life Questionnaire Core-30
Open-Label Psilocybin Study in Transdiagnostic Population	NCT06442423 Benjamin Kelmendi, MD, Yale University	1	50 patients with at least one debilitating psychiatric symptom over the last 30 d ≥18y	One arm open label, mono	1 x 25 mg psilocybin	5-HT R non-selective agonist [13]	Primary: Columbia-Suicide Severity Rating Scale; adverse events **Secondary**: 36 assessments for various psychiatric disorders including **HAMA**, drug use disorders, and personality disorders.
Psilocybin-assisted Therapy for Alcohol Use Disorder	NCT06444243 University of Sydney	2	90 patients with alcohol use disorder ≥18y	Rd, db, reference controlled, mc	2 x 25 mg Psilocybin or 2 x 250 mg niacin within 12 wks of psychotherapy	5-HT R Non-selective agonist [13]	Over 52 wks: **Primary**: Frequency of heavy drinking days **Secondary**: Mean alcohol consumption per drinking day; Phosphatidylethanol levels; WHO drinking risk level; alcohol dependence scale; Penn alcohol craving scale; **DASS-21 anxiety** and depression **scales**;

							Columbia suicide severity rating scale; short form Health Survey (SF-36); Markers of liver injury
Psilocybin-Assisted Therapy for Intergenerational Trauma	NCT06899165 Rachel Yehuda, Icahn School of Medicine at Mount Sinai	2	Child of genocide survivor with depression or anxiety ≥18y	Open label, one arm	2 doses of 25 mg psilocybin at least 3 wks apart plus weekly therapy session over 6 wks	5-HT R non-selective agonist [13]	Primary: Columbia-Suicide Severity Rating Scale; Brief Psychiatric Rating Scale (BPRS-6) **Secondary**: Change in Depression, **Anxiety,** and Stress Symptoms Scale; Change in Parental PTSD Questionnaire; Change in Resilience Scale for Adults
RE104 Safety and Efficacy Study in Adjustment Disorder in Cancer and Other Medical Illnesses	NCT07002034 Reunion Neuroscience Inc	2	100 patients with adjustment disorder with depressed mood with or without anxiety due to cancer or other serious illness 18-80 y	Rd, reference-controlled, db, pg, mc	single dose 1.5 mg or 30 mg RE104 (Luvesilocin) subcutaneous injection for 14 d; follow-up 42d	5-HT R non-selective agonist	Primary: Change in Montgomery-Åsberg Depression Rating Scale after 14 d **Secondary**: **HAM-A**; incidence of Treatment-emergent adverse event
The Role of Serotonin in Compulsive Behavior in Humans: Underlying Brain Mechanisms	NCT04336228 Rigshospitalet, Denmark	4	46 patients 18-70 y with high obsessive-compulsive traits or healthy volunteers	Rd, pg, db, pc, healthy group controlled, mono	3-4 wks 20 mg/d Escitalopram or placebo to either control people or obsessive-compulsive disorder patients	5-HT reuptake inhibition	All measures taken after 3 y: **Primary**: 24 different cognitive or psychological or MRI tests **Secondary**: 46 different cognitive or psychological tests, incl. **STAIS** Other outcome measures: 33 additional psychological tests
Treating Negative Affect in Low Back Pain Patients	NCT04747314 Ajay Wasan, MD, Msc	2/3	330 patients with >6 months lower back pain and comorbid depression or anxiety 18-75 y	Rd, open, mc	4 months not specified anti-depressant medication, or Enhanced Fear Avoidance Rehabilitation (EFAR), or a combination thereof;	5-HT, noradrenergic signaling	Baseline vs 4 months Primary: Physical function, pain, depression assessed with PROMIS short form; **Secondary**: pain interference, **anxiety**, sleep using PROMIS short form; opioid craving; patient GIC; Current Opioid

					drug non-responders continued for another 4 months with anti-depressant with or without EFAR. Some of EFAR non-responders assigned to either another 4 months of EFAR, or another 4 months anti-depressant		Misuse Measure (COMM) questionnaire
Cannabidiol Effects on Fear Extinction in Social Phobia	NCT06123702 Hartford Hospital	1 early	20 SAD patients ≥18y	Rd, db, pc, pg, mono	600 mg cannabidiol (CBD) or placebo	Cannabinoid R modulation? Interaction with other neurotransmitters?	Skin conductance, subjective units of discomfort in fear conditioning paradigm
Efficacy and Safety of Inhaled NC-107 As Compared to Placebo After 4 Weeks of Treatment in Patients with Anxiety (CALM)	NCT06656806 Trinity Hypertension & Metabolic Research Institute with Nutraceutical Corporation	1 early	40 anxiety patients 18-80 y	Non-rd, db, pc, mono	4 wks inhaled NC-107 (cannabidiol, CBD formulation) puffs bid or placebo	Cannabinoid R modulation? Interaction with other neurotransmitters?	Primary: CBD concentration in plasma **Secondary**: CBD concentration in urine; **decrease in STAI or GAD-7 questionnaires**
Cannabis For Cancer-Related Symptoms	NCT03948074 Pippa Hawley, British Columbia Cancer Agency	2	150 patients ≥19 y with cancer-related Nausea; Pain; Anxiety; Sleep Disturbance	Rd, db, pc, multiple crossover, mc	573 mg THC/THCa, or 37 mg THC/THCa+784 mg CBD/CBDA, or 516 mg THC/THCa+456 mg CBD/CBDA, or placebo	Cannabinoid R, TRP R, 5-HT_{1A} R, L-type Ca^{2+}; PPARγ [14]	**Primary**: 90 min after each dose GIC for cancer-related symptoms **Secondary**: Average change from baseline **Edmonton Symptom Assessment System** incl. sleep disturbance and night sweat symptoms Other: Preference of each study subject for type of oil
Effectiveness of Deep Brain Stimulation for	NCT00640133 Butler Hospital	4	patients with disabling OCD 18-75 y	Rd, sham controlled, pg, mc	DBS or sham DBS	Disruption of brain signal transduction [15]?	Primary: Yale-Brown Obsessive-Compulsive Scale ; Global Assessment of Functioning Scale; Social and Occupational

Treating People With Treatment Resistant Obsessive-Compulsive Disorder							Functioning Assessment Scale **Secondary**: Quality of Life Enjoyment and Satisfaction Questionnaire; Montgomery-Asberg Depression Rating Scale; Behavioral Activation for Depression Scale; **HAMA**; HAMD
Aripiprazole in Body Focused Repetitive Behaviors	NCT05545891 University of Chicago	2	100 patients with trichotillomania or skin picking disorder ≥18 y	Rd, db, pg, mono	3 wks 5 mg/d, then 6 wks 10 mg/d aripiprazole or placebo	Dopamine D2R partial agonist (reducing D2R activation), partial $5HT_{1A}$ agonist; 5-HT_{2A} antagonist	After 6 wks treatment Primary: NIMH Symptom Severity Scale for hair pulling and skin picking **Secondary**: CGI Improvement Scale; Massachusetts General Hospital Hairpulling (Skinpicking) Scale; Skin Picking Symptom Assessment Scale; Quality of Life Inventory; Sheehan Disability Scale; HAMD; **HAMA**; Tridimensional Personality Questionnaire, Cambridge Caffeine Use Survey; Cambridge-Chicago Trait Scale
An Extension Test of Whether to Use Oral Anti-anxiety Drugs (XANAX) When Patients Choose Second Eye Cataract Surgery After Unblinding, and Analyze Their Anxiety, Satisfaction	NCT06874452 National Taiwan University Hospital	4	250 patients after cataract surgery on one eye, before surgery of the other eye 18-80y	Open label, pg, mono	1 x 0.5 mg (0.25 mg for patients >65 y) alprazolam (XANAX) or placebo 30 min before surgery	$GABA_AR$	1 d after procedure **Primary**: Anesthesia satisfaction rating scale **Secondary**: **State-Trait Anxiety Inventory, Beck Anxiety Inventory; postoperative pain visual analogue scale**

and Pain Satisfaction							
Ozone Treatment in Paresthesia (Numbness, Tingling) Secondary to Chemotherapy-induced Peripheral Neuropathy	NCT06706544 Bernardino Clavo, MD, PhD	2/3	42 patients with chemotherapy-induced paresthesia ≥18 y	Rd, pg, pc, db, mono	40 x rectal ozone treatment over 16 wks or oxygen (placebo)	Immunemodulation? Neuroprotection? [17]	**Primary**, at 28 wks: change in numbness and tingling; change in QOL rating **Secondary** 28 wks: Hospital costs; neuropathy CTCAE v.5.0. and QLQ-CIPN20 scales; QOL QLQ-C30; **HADS**; serum oxidative stress and inflammation markers; hyperspectral and infrared images of hands and feet; toxicity in rectal zone CTCAE v.5.0. scale; Secondary 16 wks: numbness and tingling; neuropathy CTCAE v.5.0. and QLQ-CIPN20 scales; QOL EQ-5D-5L and QLQ-C30 questionnaire; **HADS**; ; serum oxidative stress and inflammation markers; hyperspectral and infrared images of hands and feet; toxicity in rectal zone CTCAE v.5.0. scale
Gut Microbiome Profiles in Patients with Chemotherapy-induced Neuropathy in the RCT OzoParQT (NCT06706544)	NCT06799351 Bernardino Clavo, MD, PhD	2/3	42 patients with chemotherapy-induced paresthesia ≥18 y	Rd, pg, pc, db, mono	40 x rectal ozone treatment over 16 wks or oxygen (placebo)	Immunemodulation? Neuroprotection? [17]	**Primary** at 16 wks: gut microbiome profile; numbness and tingling self-report; neuropathy according to QLQ-CIPN20 and CTCAE v.5.0. scales; **Secondary**: QOL EQ-5D-5L and QLQ-C30 questionnaires; **HADS**; serum levels of oxidative stress and inflammation biomarkers

Ventral Capsulotomy for Intractable OCD	NCT05659082 Butler Hospital	2	50 patients that will undergo capsulotomy for severe OCD 18-90 y	Single arm, mono	Capsulotomy brain surgery using laser interstitial thermal therapy	Interruption of thalamo-cortical connectivity	Primary: Yale-Brown Obsessive-Compulsive Scale (YBOCS) at 12 months and 24 months **Secondary**: **HAMA;** HAMD.
Self-Adjusted Nitrous Oxide: A Feasibility Study in the Setting of Vasectomy	NCT05895383 Beth Israel Deaconess Medical Center	4	35 vasectomy patients 21-85 y	Single group, open, mono	Self-adjusted Nitrous oxide applied via plastic mask	Nitrosylation of thiols and metalloenzymes, vasodilation, etc.	**Primary**: Visual analogue scale 0-10 for pain **Secondary**: VAS for anticipated pain; **VAS for anticipated anxiety**; VAS for procedural anxiety; VAS for recalled pain; VAS for recalled anxiety; Likert scale for satisfaction; Surgeon ease of performing questionnaire; frequency of emergent adverse events
Feasibility Study of Oral Ketamine Versus Placebo for the Treatment of Anxiety in Patients With Pancreatic Cancer	NCT05086250 Cedars-Sinai Medical Center	1 early	20 patients diagnosed with pancreatic cancer ≥18y	Rd, db, cross over, mono,	4 wks weekly oral 0.5 mg/kg ketamine or placebo, 2 wks washout, crossover	NMDAR and other R inhibition [18]	**Primary**: Feasibility measures **Secondary**: adverse events assessed per CTCAE v.5; adverse symptom checklist
Effects of Intranasal Ketamine on Depression and Anxiety in Palliative Care Cancer Patients	NCT06665568 University of Zürich	1 early	100 patients with progressive cancer and anxiety or depression ≥18 y	Single group, open label, mono	5 – 50 mg intranasal ketamine hydrochloride 8 wks	NMDAR and other R inhibition [18]	From wk 1 to wk 8, Primary: Montgomery-Asberg Depression Scale **Secondary**: **HAMA**; Quality of Life Questionnaire 30; Pittsburgh Sleep Quality Index; HADS; Zarit caregiverBurden Scale; CareGiver Oncology Quality of Life Questionnaire; Pittsburg sleep quality index

Behavioral Treatment and Memantine in Body Focused Repetitive Behaviors	NCT05796752 University of Chicago	2/3	Patients with trichotillomania (TTM) or skin picking disorder (SPD), ≥18 y	One arm, mono	2 wks 10 mg/d, then 6 wks 20 mg/d memantine; 4 wks washout; 8 wks ComB behavioral therapy	NMDAR Blockade	**Primary**: National Institutes of Mental Health Symptom Severity Scale (for trichotillomania or Skin Picking) **Secondary**: CGI-I; HAMD; **HAMA**; Massachusetts General Hospital Hairpulling Scale -- Revised for Skin Picking; Quality of Life Inventory; Sheehan Disability Scale; Barratt Impulsiveness Scale
Single-Site Study of Naltrexone/Acetaminophen for the Acute Treatment of Migraine: A Phase 2 Randomized Trial	NCT05685225 Allodynic Therapeutics, Inc	2	300 migraine patients 18-75 y	Rd, pg, pc, db, mono	Stage I: Either Naltrexone or Acetaminophen or a combination thereof or placebo. Stage II: Naltrexone plus Acetaminophen either high dose, medium dose, or low dose, or placebo during migraine attack	Opioid competitive antagonist/weak agonist (Naltrexone) COX inhibition; Cannaboid R agonist (Acteaminophen)	**Primary**: 2 h after dosing: Proportion of patients with no pain, and proportion of patients free of most bothersome symptoms **Secondary**: after treatment proportion of subjects with pain relief, freedom of photophobia, phonophobia, nausea, sustained pain relief/freedom, functional disability at 2 h and 24 h; use of rescue medication within 24 h, relapse with 48 h. **HAMA baseline vs 2 h and 24 h**; Self-reported sense of wellbeing, hurt, tension
Effects of Pioglitazone on Stress Reactivity and Alcohol Craving	NCT05107765 The University of Texas Health Science Center, Houston	1/2	60 alcohol abuse disorder patients with anxiety or stress ≥18y	Rd, pc, db, pg, mono	Initial 30 mg/d pioglitazone, increase to 45 mg/d in 1 wk, then 7 wks 45mg/d or placebo	PPARγ-mediated effect on glucose metabolism [20]	Primary: Change in Stress-reactivity assessed by Cold Pressor Task; Change in alcohol craving scale **Secondary**: Change in drinking habits; perceived stress scale; **HAMA**; change in Pennsylvania Alcohol craving scale

Anxiety and Depression Levels in Cancer Patients After Self-Application of EFT (Emotional Freedom Techniques)	NCT00737399 Soul Medicine Institute	1 early	14 patients with cholangiocarcinoma18-89 y	Single group open, mono	Emotional Freedom Techniques Emotional Freedom Techniques taught in internet and telephone group coaching	Psychotherapy	(no details provided) **Primary**: Depression **Secondary**: **Anxiety**, pain, insomnia
REmotely-delivered Supportive Programs for Improving Surgical Pain and disTrEss	NCT05625360 Wake Forest University Health Sciences	3	160 women with cancer scheduled for an abdominal uterine or ovarians surgery ≥18 y	Rd, pg, 2 centres	Either 20 min mindful breathing and movement video or life impact reflection	Psychotherapy	Primary: 4 wks postoperatively numeric pain rating scale **Secondary** at 2 wks, 4 wks, 3 months: Pain intensity rating; affective pain questionnaire; postoperative acute pain questionnaire; change in pain interference; depression, **anxiety**, sleep disturbance **short form 4 a questionnaires**
Use of a Generative AI (Gen-AI) Chatbot for Anxiety and Depression Among Persons With Cannabis Use	NCT06920238 Trustees of Dartmouth College	1	15 patients ≥18y with cannabis use disorder plus anxiety and/or depression	Single arm, mono	4 wk engagement with generative AI chatbot	Psychotherapy	**Primary**: Frequency and duration of interaction with Chatbot; System usability score; acceptability and adherence scale
Art-Based Pain Assessment Tool in Patients With Temporomandibular Joint Disorders	NCT07018791 King Abdulaziz University	3	70 patients with temporomandibular Disorders (TMD) requiring conservative management only ≥18 y	Rd, pg, diagnostic, mono	Patients are shown 3 paintings and asked which one resonates best with them, to understand patient pain experience, or only standard treatment	Psychotherapy	**Primary**: Patient pain understanding and reporting tool **Secondary**: VAS Pain level; various measures of temporomandibular joint function; **GAD-7**; PHQ-9
Project EMPOWER-OCD	NCT07158801 Boston University Charles River Campus	1	110 caregivers for people with obsessive-	Rd, db, mono	Web-based 20-25 min intervention	Psychotherapy	Primary after 2 wks and 4 wks: Family accommodation scale

			compulsive disorder ≥18 y		program or another psychoeducation		change from baseline; children's Yale-Brown obsessive-compulsive scale<br>Secondary: Parental stress scale; program feedback scale; perceived pre-to-post intervention change
Aging and Reward System Response to Inflammation and Anxiety Study	NCT05363527 University of California, Los Angeles	1	40 patients, 30 of them with anxiety, 10 with low anxiety 60-80y	Rd, pc, pg, db, mono	0.8 ng/kg body weight endotoxin or placebo	Systemic inflammation	Mostly 2 h post injection: **Primary**: Monetary Incentive Delay Task assessed via fMRI in ventral striatum; Effort Expenditure for Rewards Task assessed via fMRI in ventral striatum, ventromedial prefrontal cortex; fMRI of ventral striatum and ventromedial prefrontal cortex during receipt of money reward and in a positive picture viewing task<br>**Secondary**: 11 different tests mostly related to reward and motivation pathways

Abbreviations: ASEC: Antidepressant side effect checklist; BAI Beck Anxiety Inventory self-rated score; Bid: twice per day; CGII scale: Clinical Global Impression – Improvement scale; CGI-S Clinical Global Impression - Severity scale; GIC global impression of change; Co: cross-over; Db: double-blind; dd: double-dummie; GAD: Generalized Anxiety disorder; HADS: Hospital anxiety and depression anxiety sub-score.; HAMA: Hamilton Anxiety Rating Scale; HAMD: Hamilton Depression Rating scale; Mc multicenter; Mono: monocentric; OCD: Obsessive-compulsive disorder; open: open label; Pc: Placebo controlled; PCL: PTSD checklist; Pg: parallel groups; Pk: pharmacokinetic; PSWQ Penn State Worry Questionnaire; PTSD: Post-traumatic stress disorder; QOL: quality of life; R: receptor; Rd: randomized; SAD: social anxiety disorder; SADC: separation anxiety disorder of childhood; Sc: sham control; SNRI: Serotonin/noradrenaline reuptake inhibitor; SSRI: Serotonin uptake inhibitor; STAI: State-Trait Anxiety Inventory; VAS: Visual analogue scale; Wk: Week
