# Supplementary material for: Current Clinical Trials to Treat Anxiety Disorders in the Elderly: A Registry-Based Review
Source: Pharmaceuticals (Basel). 2026 Jun 4;19(6):891. doi: 10.3390/ph19060891 (PMC13305826; doi:10.3390/ph19060891)
Supplement: Supplementary file 1 [file pharmaceuticals-19-00891-s001.zip › Table S3 Excluded trials.pdf]

Supplementary Table S3. Trials selected in *clinicaltrials.gov* using our search query that were excluded from the analysis of examining anxiety in the elderly.

Study title, Link	Trial ID	Reason for exclusion
The Effects of Ginseng on Cancer-Related Fatigue	NCT0137511	Anxiety not specifically assessed
Neurocircuitry of Obsessive-Compulsive Disorder: Modulation by Transcranial Magnetic Stimulation	NCT02704117	Anxiety not specifically assessed
Outcomes Mandate National Integration With Cannabis as Medicine	NCT03944447	Anxiety not specifically assessed
Cannabis For Cancer-Related Symptoms	NCT03948074	Anxiety not specifically assessed
Behavioral Exposure for Introceptive Tolerance RCT	NCT05398276	Anxiety not specifically assessed
Combination Therapy for Treatment of Sleep Disturbance in Patients With Advanced Cancer	NCT05474846	Anxiety not specifically assessed
Evaluating Buspirone to Treat Opioid Withdrawal	NCT05511909	Anxiety not specifically assessed
Clinical Trial on Agitation in Alzheimer's Dementia	NCT05543681	Anxiety not specifically assessed
Neuroactive Steroid to Treat Depressed Mood: A Trial for People With HIV	NCT05570812	Anxiety not specifically assessed
MDMA-Assisted CBT for OCD (MDMA-CBT4OCD Study)	NCT05783817	Anxiety not specifically assessed
Community-based Smoking Cessation Treatment for Adults With High Stress Sensitivity	NCT06053567	Anxiety not specifically assessed
Establishing Mobile Transcranial Magnetic Stimulation	NCT06748209	Anxiety not specifically assessed
Nebulized Ketamine for the Treatment of Major Depressive Disorder in an Inpatient Setting	NCT06752759	Anxiety not specifically assessed
Effect of Dolutegravir Compared with Darunavir/Cobicistat on the Severity of Neuropsychiatric Effects Al 12 Weeks in Antirretroviral Treatment-Naive Adults.	NCT06787976	Anxiety not specifically assessed
TRIPS - Treatment to Improve Depression and/or Anxiety Using Psilocybin-assisted Psychotherapy in Cancer Survivors	NCT06801041	Anxiety not specifically assessed
Roxadustat for Bone and Neuropsychiatric Aspects in Hemodialysis Patients	NCT06917950	Anxiety not specifically assessed
Near Infrared Transcranial Laser Therapy Subjects With Major Depressive Disorder	NCT06934135	Anxiety not specifically assessed
**Psilocybin for Treatment of OCD-2**	NCT06992999 Francisco A Moreno, University of Arizona	Anxiety not specifically assessed
Psychoneuromentalism Disorder: A Medical Condition That Affects People With Psychological Impairments From Health Issues	NCT06235177	Endpoints insufficiently clear

Investigating the Protective Impact of LY-2940094 on Stress-induced Depression- and Anxiety-related Phenotypes in Humans	NCT06786637	Endpoints only include baseline assessment
From Prevention to Treatment: How Biological Rhythms Can Maintain Perinatal Mental Health	NCT06246214	Only female patients 4-6 wks postpartum
A Dyadic Approach to Perinatal Depression in Primary Care: Maternal Infant and Dyadic Care	NCT04998721	Only pregnant women included
Family Acceptance Project Online (Pilot RCT)	NCT06839859	Patients >20 y excluded
Study of Neuro-Cognitive Correlates of Pediatric Anxiety Disorders	NCT00018057	Patients >65 excluded
Understanding How Ketamine Brings About Rapid Improvement in OCD	NCT02624596	Patients >65 excluded
Troriluzole (BHV-4157) in Adult Participants With Obsessive Compulsive Disorder	NCT03299166	Patients >65 excluded
Efficacy of Nitrous Oxide in OCD: Pilot Study	NCT03826693	Patients >65 excluded
Brain Network Changes Accompanying and Predicting Responses to Pharmacotherapy in OCD	NCT04131829	Patients >65 excluded
Pragmatic Trial of Obsessive-compulsive Disorder	NCT04539951	Patients >65 excluded
Efficacy and Safety Study of Adjunctive Troriluzole in Obsessive Compulsive Disorder	NCT04641143	Patients >65 excluded
Efficacy and Safety Study of Adjunctive Troriluzule in Obsessive Compulsive Disorder	NCT04693351	Patients >65 excluded
Long-term Safety Study of Adjunctive Troriluzole in Subjects With Obsessive Compulsive Disorder	NCT04708834	Patients >65 excluded
Evaluating the Feasibility, Safety and Efficacy of Psychotherapy Assisted Psilocybin for Treatment of Severe OCD	NCT04882839	Patients >65 excluded
Study of Dextromethorphan in OCD and Related Disorders	NCT04899687	Patients >65 excluded
Epidiolex in Obsessive Compulsive Disorder and Related Disorders	NCT04978428	Patients >65 excluded
The Impact of Focused Ultrasound Thalamotomy of the Anterior Nucleus for Focal-Onset Epilepsy on Anxiety	NCT05032105	Patients >65 excluded
Social Anxiety MDMA-Assisted Therapy Investigation	NCT05138068	Patients >65 excluded
iTBS-DCS in Obsessive Compulsive Disorder	NCT05177601	Patients >65 excluded
Efficacy of Valbenazine for the Treatment of Trichotillomania in Adults	NCT05207085	Patients >65 excluded
Effects of Repeated Psilocybin Dosing in OCD	NCT05370911	Patients >65 excluded

Assessing the Impact of Cannabidiol for Anxiety and Depression in Bipolar Disorder	NCT05457465	Patients >65 excluded
Resources, Inspiration, Support and Empowerment (RISE) for Black Pregnant Women	NCT05552053	Patients >65 excluded
A Clinical Trial of Tolcapone in Obsessive Compulsive Disorder	NCT05624528	Patients >65 excluded
A Clinical Trial of Fecal Microbiota Transplantation in a Population With Obsessive-compulsive Disorder	NCT05720793	Patients >65 excluded
A Clinical Study That Will Measure How Well SEP-363856 Works and How Safe it is in Adults With Generalized Anxiety Disorder	NCT05729373	Patients >65 excluded
Effect of SPG Block for Patients With Anxiety at Electronic Dance Music Festivals	NCT05729503	Patients >65 excluded
ENCALM: a Study of ENX-102 As a Monotherapy Treatment in Patients with Generalized Anxiety Disorder	NCT05749055	Patients >65 excluded
Clinical Trial of Shugan Jieyu Capsule in Generalized Anxiety Disorder	NCT05772104	Patients >65 excluded
Examining Mu Opioid Mechanisms of Ketamine's Rapid Effects in OCD (MKET2)	NCT05940324	Patients >65 excluded
Effects of Melissa Extract on Sleep Characteristics	NCT0595093	Patients >65 excluded
A Study to Evaluate Efficacy and Safety of Toludesvenlafaxine Hydrochloride Sustained-release Tablets in Generalized Anxiety Disorder	NCT05970510	Patients >65 excluded
Processes and Circuitry Underlying Threat Sensitivity as a Treatment Target for Co-morbid Anxiety and Depression	NCT06004115	Patients >65 excluded
A Study of a N, N-dimethyltryptamine (DMT) Analog (CYB004) in Participants with Generalized Anxiety Disorder (GAD)	NCT06051721	Patients >65 excluded
Personalized Brain Stimulation to Treat Chronic Concussive Symptoms	NCT06073886	Patients >65 excluded
A New Intervention to Improve Function in Veterans With Anxiety and Depression	NCT06188923	Patients >65 excluded
Neural Circuit Effects of Ketamine in Depression	NCT06213324	Patients >65 excluded
Adjunctive Treatment With L-methylfolate for Treatment-Resistant Generalized Anxiety Disorder: a Pilot Study	NCT06218030	Patients >65 excluded
Phase Ⅲ Clinical Trial of Buagafuran Capsules in the Treatment of GAD **(BGFN-2022-01)**	NCT06243614	Patients >65 excluded

Phase 3 Clinical Trial of Buagafuran Capsules in the Treatment of GAD	NCT06243640	Patients >65 excluded
Testing a Transdiagnostic TMS Treatment Target	NCT06282146	Patients >65 excluded
Feasibility, Clinical Effects, and Safety of Psilocybin-assisted Psychotherapy for Treatment-resistant OCD	NCT06299319	Patients >65 excluded
Fasedienol Nasal Spray for the Acute Treatment of Anxiety in Adults with Social Anxiety Disorder (PALISADE-3)	NCT06358651	Patients >65 excluded
Search for Novel Transcranial Magnetic Stimulation (TMS) Targets for Mental Illness	NCT06376734	Patients >65 excluded
Efficacy and Safety of HB-1 for Panic Disorder	NCT06483789	Patients >65 excluded
Efficacy of BNC210 in Acute, As-needed Treatment of Anxiety in Social Anxiety Disorder - 1	NCT06510504	Patients >65 excluded
Autonomous Digital CBT Intervention for Opioid Use Disorder in Individuals With Co-Occurring Internalizing Disorders	NCT06545071	Patients >65 excluded
Pilot Study of RR-HNK in OCD	NCT06575075	Patients >65 excluded
Fasedienol Nasal Spray for the Acute Treatment of Anxiety in Adults With Social Anxiety Disorder (PALISADE-4)	NCT06615557	Patients >65 excluded
Evaluating the Efficacy and Safety of PROSOMNIA Sleep Therapy™ in Patients With Sleep Deprivation and Chronic Insomnia	NCT06644573	Patients >65 excluded
A Dose-Response Safety Study of ENX-102 in Patients With GAD	NCT06653296	Patients >65 excluded
Exploratory Safety and Efficacy of EMP-01 in Social Anxiety Disorder	NCT06693609	Patients >65 excluded
A Study of the Safety and Exploratory Efficacy of Oral AFA-281 in Patients with Alcohol Use Disorder	NCT06710431	Patients >65 excluded
Effect of Probiotics "Psychobiotics" on Depression and Metabolic Syndrome in Saudi Arabia	NCT06765057	Patients >65 excluded
A Basket Clinical Study to Assess Glycerol Tributyrate in Patients With Mitochondrial Encephalopathy, Lactic Acidosis, Stroke-like Episodes (MELAS) or Leber's Hereditary Optic Neuropathy-Plus (LHON-Plus)	NCT06792500	Patients >65 excluded
A Study of ONO-1110 in Patients With Social Anxiety Disorder	NCT06805565	Patients >65 excluded

A U.S. Double-blind, Placebo-controlled Phase 2 Clinical Trial to Assess the Efficacy, Safety, and Tolerability of a Repeat Dose of Fasedienol Nasal Spray for the Acute Treatment of Anxiety in Adults with Social Anxiety Disorder	NCT06809179	Patients >65 excluded
A Mechanistic Study to Assess a Single Dose of CYB003 in Participants with Depression and Anxiety	NCT06820723	Patients >65 excluded
Priming CBT with RTMS for OCD	NCT06840951	Patients >65 excluded
Study to Assess Adverse Events and Change in Disease Activity When Oral ABBV-932 is Added to Antidepressant Therapies in Adult Participants With Generalized Anxiety Disorder	NCT06846320	Patients >65 excluded
Promoting Active Therapy: Path to Wellness	NCT06858189	Patients >65 y excluded
A Trial of Centanafadine Efficacy and Safety in Adults With Attention-deficit/Hyperactivity Disorder and Comorbid Anxiety	NCT06973577	Patients >65 y excluded
The Combination of Pharmacotherapy and Cognitive Behavioral Psychotherapy Under the Recovery Perspective.	NCT06993662	Patients >65 y excluded
Integrating Inuit Knowledge Principles in Multi-Level Mental Health Clinical Trials	NCT07014852	Patients >65 y excluded
A Study to Evaluate the Efficacy and Safety of SPT-300 (GlyphAllo) in Participants With Major Depressive Disorder, With or Without Anxious Distress (BUOY-1 Study)	NCT07065240	Patients >65 y excluded
Magnetic Resonance-Guided Focused Ultrasound Bilateral Capsulotomy for Refractory Anorexia Nervosa With Comorbid Obsessive Compulsive Disorder or Major Depressive Disorder	NCT07113665	Patients >65 y excluded
Study to Evaluate the Efficacy and Safety of VQW-765 for the On-Demand Treatment of Social Anxiety Disorder	NCT07221578	Patients >65 y excluded
